# Basic practical skills teaching and learning in undergraduate medical education – a review on methodological evidence

**DOI:** 10.3205/zma001063

**Published:** 2016-08-15

**Authors:** Daniela Vogel, Sigrid Harendza

**Affiliations:** 1 Universitätsklinikum Hamburg-Eppendorf, III. Medizinische Klinik, Hamburg, Deutschland

**Keywords:** basic practical skills, clinical skills, physical examination, skills training, undergraduate medical education

## Abstract

**Objective: **Practical skills are an essential part of physicians’ daily routine. Nevertheless, medical graduates’ performance of basic skills is often below the expected level. This review aims to identify and summarize teaching approaches of basic practical skills in undergraduate medical education which provide evidence with respect to effective students’ learning of these skills.

**Methods: **Basic practical skills were defined as basic physical examination skills, routine skills which get better with practice, and skills which are also performed by nurses. We searched PubMed with different terms describing these basic practical skills. In total, 3467 identified publications were screened and 205 articles were eventually reviewed for eligibility.

**Results: **43 studies that included at least one basic practical skill, a comparison of two groups of undergraduate medical students and effects on students’ performance were analyzed. Seven basic practical skills and 15 different teaching methods could be identified. The most consistent results with respect to effective teaching and acquisition of basic practical skills were found for structured skills training, feedback, and self-directed learning. Simulation was effective with specific teaching methods and in several studies no differences in teaching effects were detected between expert or peer instructors. Multimedia instruction, when used in the right setting, also showed beneficial effects for basic practical skills learning.

**Conclusion: **A combination of voluntary or obligatory self-study with multimedia applications like video clips in combination with a structured program including the possibility for individual exercise with personal feedback by peers or teachers might provide a good learning opportunity for basic practical skills.

## Zusammenfassung

**Zielsetzung: **Praktische Fertigkeiten sind ein wesentlicher Bestandteil des ärztlichen Arbeitsalltags. Dennoch liegt die Leistung von Absolventen eines Medizinstudiums bei der Durchführung praktischer Basisfertigkeiten häufig unter den erwarteten Anforderungen. Diese Übersichtsarbeit verfolgt daher das Ziel, Lehrmethoden für medizinische Basisfertigkeiten im Medizinstudium zu identifizieren und zusammenzufassen, die einen evidenzbasierten Nachweis für das effektive studentische Lernen dieser Fertigkeiten erbringen.

**Methoden: **Praktische Basisfertigkeiten wurden als Basisfertigkeiten der körperlichen Untersuchung, Routinefertigkeiten, die im Laufe der Praxis besser werden, und als Fertigkeiten, die auch vom Pflegepersonal übernommen werden, definiert. PubMed wurde mit verschiedenen Begriffen durchsucht, die diese praktischen Basisfertigkeiten beschreiben. Insgesamt wurden 3467 identifizierte Publikationen gesichtet und 205 wurden schließlich auf ihre Eignung geprüft.

**Ergebnisse:** 43 Studien, die mindestens eine praktische Basisfertigkeit, einen Vergleich zweier Gruppen von Medizinstudierenden und Wirkungen auf die studentische Leistung beinhalteten, wurden analysiert. Sieben praktische Basisfertigkeiten und 15 verschiedene Lehrmethoden konnten identifiziert werden. Die konsistentesten Ergebnisse in Bezug auf effektive Lehre und den Erwerb von praktischen Basisfertigkeiten wurden für strukturiertes Fertigkeitentraining, Feedback und selbstgesteuertes Lernen gefunden. Simulation war mit spezifischen Lehrmethoden wirksam und in mehreren Studien fanden sich keine Unterschiede in Bezug auf Lehreffekte zwischen Experten oder Peers als Lehrende. Multimedia-gestützte Instruktion zeigte bei Anwendung in geeignetem Rahmen ebenfalls positive Effekte für das Erlernen praktischer Basisfertigkeiten.

**Fazit: **Eine Kombination von freiwilligem oder obligatorischem Selbststudium mit Multimedia-gestützten Anwendungen wie Video-Clips in Kombination mit einem strukturierten Programm, das die Möglichkeit für individuelle Übungen mit persönlichem Feedback von Peers oder Lehrenden beinhaltet, könnte eine gute Möglichkeit für das Erlernen praktischer Basisfertigkeiten bieten.

## Introduction

During undergraduate medical education knowledge, skills, and attitudes have to be acquired by medical students to provide competent patient care after graduation. The term “skills” often comprises communication skills, physical examination skills, practical skills, psychomotor skills, clinical skills, technical skills and others without further specification. A current approach in health profession education is the development of competence-based undergraduate curricula [56]. In Germany, a National Competence Based Catalogue of Learning Objective for Undergraduate Medical Education (NKLM) came into effect in June 2015 [10]. Many of the competences described in the NKLM include the acquisition of basic practical skills [http://www.nklm.de [accessed 19.9.2015]]. 

Regarding basic practical skills (i.e. accomplishing a task like knot tying or cardiac auscultation) [http://curriculum.racgp.org.au/media/12371/proceduralskills.pdf [accessed 19.9.2015]], complaints have been raised by medical stakeholders that medical graduates execute such skills below the expected level of performance [43]. Furthermore, third year undergraduate medical students reported their competence in core clinical skills like rectal examination or insertion of a nasogastric tube on average with 4.7 on a 6-point (1=excellent) Likert scale [11]. On the other hand, different basic clinical skills training programs seem to offer medical students different levels of preparedness with respect to physical diagnostic skills [42], suggesting that some teaching methods for practical skills might result in better performance. An obstacle for teaching practical skills well has been identified in some teachers’ lack of confidence in their own physical examination skills [38].

To develop or remodel an undergraduate medical curriculum with the goal of being competency-based, optimal and effective teaching strategies how to acquire basic practical skills need to be implemented. This review aims to identify and summarize teaching approaches of basic practical skills in undergraduate medical education, which provide evidence with respect to student learning of these skills.

## Methods

### Basic practical skills

No unanimous term is used in medical education literature to describe basic practical skills. Neither is there a unique definition, which skills can be summarized under basic practical skills. The terms procedural skills, (basic) surgical skills, physical examination skills, (basic) clinical skills, hands-on skills, basic skills, technical skills, elementary techniques, motor skills, (basic) surgical techniques, psychomotor skills, psychomotor task, clinical technical skills, manual tasks, elementary procedures and physical diagnosis, and basic technical procedures are used inconsistently to describe similar or overlapping practical skills including either aspects of physical examination or procedures involving medical instruments, resembling the technical dimension of professional competence by Epstein and Hundert [8].

In 2011, the GMA Committee on Practical Skills published a consensus statement on 289 practical skills in undergraduate medical education [49]. Mastery of the different skills should either be achieved by medical students before starting clerkships, final year training or internship and the different levels of teaching and learning for the individual skill were defined as having watched a teacher while performing the respective skill, having conducted the skill oneself under supervision or being able to use the skill appropriately to the situation by oneself [49]. Based on the highest levels of this classification and excluding communication, emergency, and soft skills, skills included in this review were defined as being basic practical skills when they matched one of the following criteria:

- physical examination skills which every student should be able to perform independently of the intended postgraduate training (e.g. cardiac auscultation),
- simple routine medical skills which get better with practice (e.g. venipuncture),
- practical skills, which are also performed by nurses (e.g. bladder catheterization).

#### Selection criteria

We wished to identify studies that described

1. teaching methods for any of the basic practical skills described in the definition above and
2. provided evidence that the respective teaching methods showed an effect on students’ performance of the respective skill.

#### Strategy of literature search

Since a clear definition for basic practical skills is lacking, we searched PubMed using the search term “medical education” in combination with either “basic skills”, “basic technical procedures”, “clinical skills”, “clinical technical skills”, “hands-on skills”, “master learning”, “motor skills”, “physical examination skills”, “practical skills”, “procedural skills”, “psychomotor skills”, “surgical skills”, “surgical techniques”, or “technical skills” for articles in either English or German published between January 2000 and September 2015. The volumes 2000 to 2010 of the GMS Zeitschrift für Medizinische Ausbildung that are not listed in PubMed were searched individually.

This original compiled search resulted in a total of 3467 publications. For further consideration, only full research articles with undergraduate medical students being the studies’ subjects were included; short papers, letters or comments were excluded. In this step all titles and abstracts were screened and only manuscripts including at least one of the desired basic practical skills were included for further screening, resulting in 205 articles. Duplicates were also excluded in this step. These 205 manuscripts were subdivided in the following categories: controlled studies, theoretical advices and reviews, evaluation studies and surveys, pre-post studies, observational studies, and qualitative studies.

## Results

**Figure 1:**
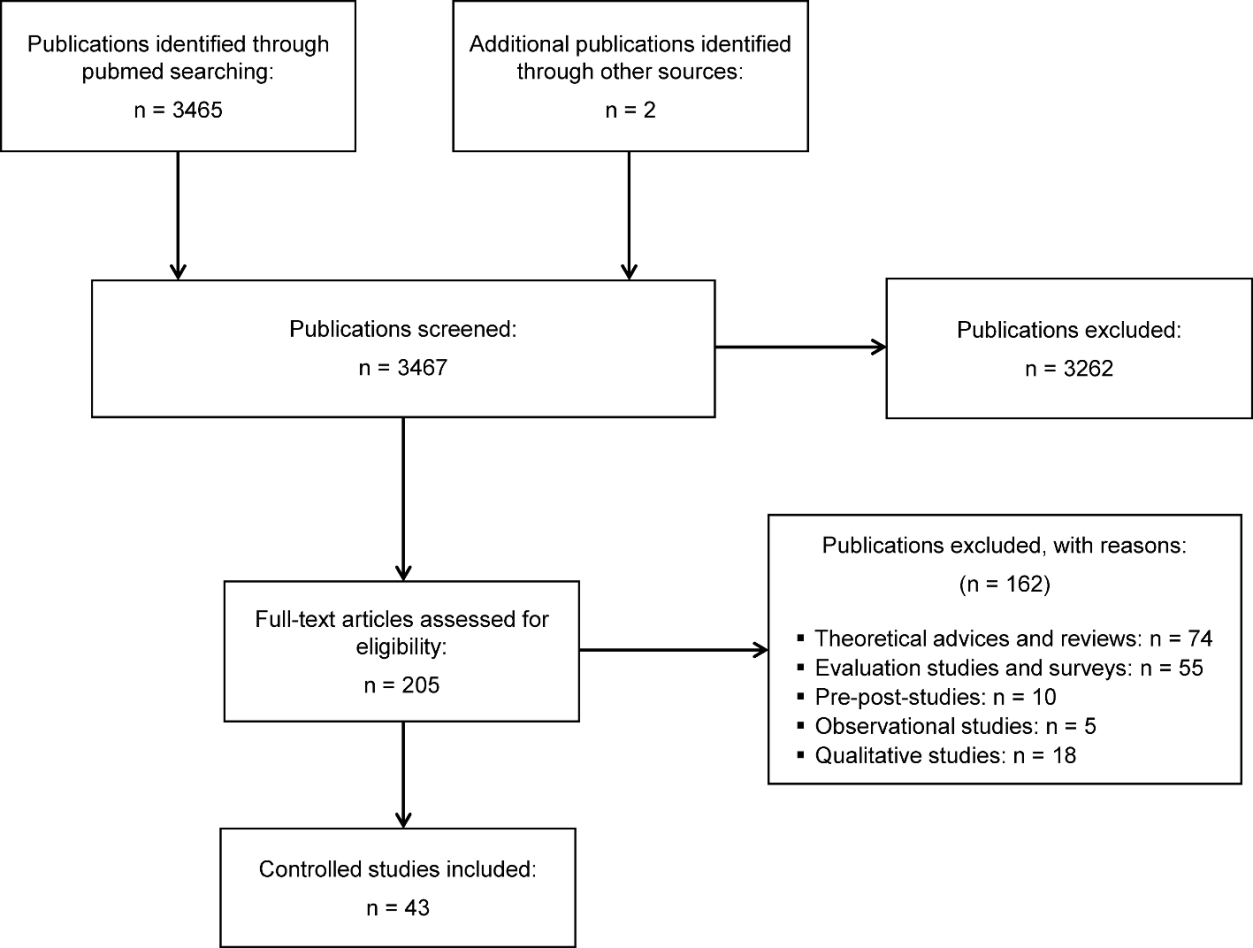
Strategy of literature search and study selection

Manuscripts matching the different categories are displayed in Figure 1 (Fig. 1). We identified 43 publications as controlled studies including medical students and at least one basic skill matching the selection criteria, comparing at least two groups and using an assessment to measure skill performance. Table 1 (Tab. 1) shows the different basic practical skills covered by the 43 publications and specifies their respective teaching or learning methods. The number of papers attributed to a specific teaching or learning method is shown in Table 2 (Tab. 2).

### Structured Skills Training

In general, student participation in structured skills training is associated with improved assessment outcomes - with respect to physical examination compared to students just following a clerkship [15] and with respect to injection and suturing skills compared to students not participating in a specific training program [26]. Different types of structured skills training programs have been developed leading to different outcomes with respect to students’ performance regarding physical examination skills or suturing. A structured bedside training where attendings received guidelines to demonstrate and observe students doing physical examinations, led to better student performance in half of the OSCE stations covering heart and lung examination [44], while another study with specific weekly bedside instructions in physical examination skills compared with the usual bedside teaching showed better OSCE results for musculoskeletal, pulmonary and gastrointestinal but not for cardiovascular exam [50].

Students being taught physical examination in a clinical coaching program with weekly structured teaching by paid general practitioners showed better OSCE results than students receiving weekly opportunistic teaching by unpaid hospital-based specialists without specific feedback [39]. Training in a skills lab with a set of specific exercises on dummies or paired peers versus standard bedside teaching was associated with better OSCE scores for abdominal examination but not for cardiac auscultation [20]. Another study reporting on an obligatory training program in a skills laboratory where students can practice skills on each other, on models, manikins, and standardized patients, shows better OSCE results for students participating in this program versus students from a traditional curriculum for lung and heart examination but not for examination of the abdomen and for injection and suturing techniques [33]. With respect to the latter, a specific surgical skills training workshop series demonstrated significant improvement for suturing skills [28], while another study identified the optimal instructor : student ratio to be one instructor for four students [7].

#### Different instructors

The question, who might be the optimal instructor for clinical skills teaching, is addressed in nine controlled studies. For physical examination skills there is no difference in OSCE results between students taught by peers versus physicians [18], generalists versus specialists [63] or standardized physical examination teaching associates versus faculty [1]. Better OSCE results were reported for students taught by full-time faculty versus part-time faculty [64]. Regarding suturing skills, peer teaching and faculty teaching lead to equal practical test results [6] and there was also no difference between being taught by a non-surgical skills coach versus being taught by a surgeon [23]. For injections skills, peer-teaching lead to similar student skills like faculty teaching [59]. For bladder catheterization, one study showed equal results for students being taught by peers or faculty [55], while another study showed better performance of students being taught by experts [58]. 

#### Multimedia-assisted instruction

With respect to multimedia-assisted instruction different aspects of the application of multimedia have been studied. Students who had access to standard video clips for different aspects of physical examination received better OSCE results than a cohort of students studying without being given this learning opportunity [32]. Students who learned with the “click-version” of an interactive program of abdominal exam performed better than students who worked with the “drag-version” of the same program [21]. When cardiac auscultation was learned with a CD-ROM in addition to the usual clinic rotation, auscultation skills significantly improved and this improvement lasted even until one year after the intervention [52]. Concerning suturing skills, self-study with interactive video instruction lead to similar performance than self-study with video and expert instruction [31]. Feedback including the possibility to watch one’s own performance on a video was associated with better suturing skills than verbal feedback alone [9]. Working alone with an interactive video on suturing skills was associated with better performance than working in student tandems with the same video [45]. With respect to different kinds of videos teaching suturing performance was best when students were shown videos with the correct task and videos explaining errors [46]. For bladder catheterization, computer-assisted learning was as effective as expert feedback in a simulation setting [58].

#### Simulation

Students who received training of heart sounds with a high-fidelity simulator (Harvey) did not perform significantly better than students who trained with a low-fidelity simulator (CD) [5]. Students who were given the opportunity to train cardiac examination skills on standardized patients and a cardiac simulator (Harvey) performed significantly better in cardiac skills than a control group, who only worked with standardized patients [22]. Training of abdominal examination with standardized patients lead to better student performance in this examination skill than a lecture alone [12]. Training with a manikin (Laerdal SimMan 3G) resulted in better chest examination skills than performing chest examination on a peer [53]. With respect to gastric-tube insertion, being involved in role-play skills lab sessions did not result in better technical performance of this skill [30]. Simulator skills lab training for cannulation, venipuncture, and injection resulted in better performance of these skills compared to not having received simulator training [59]. Practicing injection skills on a manikin compared to another group who received additional training using a fellow student as surrogate patient did not lead to any differences with respect to the technical performance of an injection [4].

#### Feedback, self-guided learning and voluntary training

Feedback has been identified as an important method to improve the learning of skills. One of the structured weekly programs for physical examination skills described above included ongoing formative assessment and feedback by the instructors for the students, who eventually showed better OSCE performance [39]. For suturing skills, different aspects of feedback – besides watching a video with one’s individual performance [9] – for this specific skill acquisition have been studied. Verbal feedback from an expert that is adapted to the personal situation of the learner was more effective than self-accessed computer generated feedback for suturing performance [34]. Furthermore, real-time feedback with an apparatus measuring the force applied by the learner’s hand while tying a knot led to an appropriate decrease of the force needed for this sensitive task compared to a group without this specific feedback [35]. While one study showed that suture training with feedback lead to better suturing skills than self-directed suture training [6], other studies reported that self-guided suturing practice [3] or a self-directed schedule for suturing practice [47] were associated with better suturing skill acquisition and additional expert feedback lead to no further skill improvement [31]. Furthermore, voluntary participation in reflective writing and skills practice [54] and voluntary practice with positively deviant peers [62] led to better clinical skill performance of participating students.

#### Other teaching/learning methods

Observing peers performing a physical examination was associated with better student performance in an assessment of physical examination skills than just receiving feedback from a patient instructor [27]. Using ultrasonography in learning clinical examination showed some improvement for correct lung and liver palpation but not for thyroid palpation [13]. Being taught knot-tying with the kinesthetic method has led to significant better performance of this task by novices compared to medical students, who watched a traditional video [17]. Furthermore, working with process goals while learning suturing leads to greater skill retention than working with outcome goals [3]. In addition, mental imagery technique appeared to transfer learning better from practice suturing sessions to actual surgical assessment than textbook study [48]. Better cannulation skills could be demonstrated by students using cumulative sum charts to log their cannulation attempts during their finial year [51]. When students were taught gastric-tube insertion with Peyton’s Four-Step Approach as teaching method they did not differ from their peers, who received standard instruction in terms of correct stepwise performance but sored better in global rating assessing professionalism [25].

## Discussion

Many different variables have been identified from controlled studies to influence students’ learning of basic practical skills ranging from more global factors like structured skills training, multimedia-assisted instruction or different instructors to specific teaching methods like feedback, mental imagery or Peyton’s Four-Step Approach. Besides very heterogeneous teaching methods, the teaching itself was applied in different phases of the undergraduate medical curriculum from first year to final year. Depending on the context of the study the influence of the same variable can lead to different results making it all the more difficult to give comprehensive recommendations which is the best method to teach which basic practical skill. One recommendation that can be given unrestrictedly is, that providing a skills training of any sort seems to lead to better skills learning in undergraduate medical education than just participating in usually unstructured clerkships or bedside teaching [15], [20], [50]. Whether the optimal instructor : student ratio (1:4) that was identified for learning suturing [7] will also be optimal for, e.g. learning cannulation, can still not be answered. Interestingly, when post-graduate year (PGY)-1 residents and graduating PGY-3 residents where compared in an OSCE on basic practical skills, a significant increase in skills was only seen between PGY-1 week 0 and PGY-1 week 4 residents, but not between the latter and PGY-3 residents [60].

When it comes to the question who is supposed to be the instructor for a certain basic skill, the recommendation that the most skilled clinicians should be recruited to teach physical examination [36] cannot be followed uncontradictedly. Several studies showed that being taught by instructed peers or trained personnel, generalists or specialists, leads to similar skill performance in medical students [1], [6], [18], [23], [59], [63]. However, one study demonstrated that students showed better performance of bladder catheterization after being taught by an expert [58] and students who had been taught by full-time faculty performed better than students who were taught by part-time faculty [64]. With the majority of studies providing evidence that basic practical skills can be taught equally well by educated peers or non-physicians we believe that the recommendation to recruit personnel other than physicians to teach basic practical skills can be given. This recommendation refers merely to the technical part of the teaching which was in the focus of this review. However, a more competence-based approach to undergraduate medical education, which the NKLM aims for, will eventually need the integration of physical examination skills with communication and clinical reasoning, which might require medical experts as role models to teach the students [14].

With respect to practical skills, which are also performed by nurses, injections, bladder catheterization, and gastric tube insertion were learned equally well when taught by peers compared to experienced faculty staff in a skills lab [59]. Meanwhile, 10 medical faculties in Germany have a peer-assisted learning program for gastric tube insertion in their skills lab [2]. On the other hand, these skills could be learned during the three months of nursing practice every undergraduate medical student in Germany has to complete [http://www.gesetze-im-internet.de/bundesrecht/_appro_2002/gesamt.pdf [accessed 28.9.2015]]. However, it can be assumed that nursing practice for medical students is a mix of many unstructured educational events like clerkships which have been identified in a focus group study to provide no real opportunity to train skills [40]. A possible structured learning approach for venipuncture, bladder catheterization etc. could be a multiprofessional program for medical and nursing students which showed a significant increase in self-assessed confidence levels for the taught skill in a pre-post evaluation [57].

In times of tight budgets, the result that a CD-ROM worked as well to learn cardiac auscultation as the much more expensive high-fidelity simulator Harvey [5] seems to be as welcome as the finding that such a CD in addition to a clinic rotation led to better and sustainable auscultation skills [52]. Since all medical faculties in Germany provide skills lab training as part of their undergraduate medical education [2], this might be the place were learning with CDs could be integrated in auscultation exercises. How students should work with multimedia, e.g. in teams or by themselves, might depend on the type of skill they are supposed to learn. For suturing, better performance was observed when students worked individually with an interactive video [45]. For more complex tasks involving competences like clinical reasoning, having worked with a tandem partner on virtual patient cases led to better results in a knowledge test [19]. Hence, studies on individual work or teamwork with multimedia cannot be generalized. Furthermore, when designing multimedia tools for studying skills great care has to be taken to choose the best application to enhance learning. When students learned with a “drag version” of an interactive program of abdominal exam they performed worse than their peers who had used the simpler “click-version” of the same program [21]. The difference might have been due to possible cognitive overload generated by dragging items on the screen rather than simplifying the task without decontextualizing it to adapt the intrinsic load of learning the specific skill to the developmental state of the learner [61].

Feedback in general and self-directed learning in the sense of organizing one’s own schedule, being aware which skill to practice, or participating in clearly defined voluntary exercises have been identified in this review to be beneficial for acquiring different basic practical skills. These findings are in line with the results that – moving away from the ‘see-one-do-one-teach-one’ approach – structured programs support basic skill learning and might solve the complaints that unstructured electives cannot be relied upon to provide students with practical skills learning [41]. Another study among German undergraduate medical students showed, that during electives procedures are often practiced unsupervised and students might acquire incorrect techniques in the absence of feedback [11]. Feedback is recommended in the clinical environment to provide learners with information about their performance for potential improvement and as guidance for students to reassess their attainment of goals [37], which can further support self-directed learning. An additional form of feedback, i.e. observing peers [27], has been found to be associated with better student learning than just the instructor’s feedback alone. Last, but not least, very special haptic feedback methods for practical skills like suturing [35] complete the list to underscore the importance of feedback as a teaching method of basic practical skills. Essential for feedback is the direct observation of the student by the instructor which is especially challenging in the current clinical environment and supported by practical tips [16]. However, for certain basic practical skills, peer teachers [6] and non-physician educators [23] seem to serve equally well as substitute instructors.

With respect to applying specific teaching methods for learning basic practical skills, Peyton’s Four-Step Approach has been demonstrated to be effective for teaching gastric tube insertion using a manikin [25]. In another study on practicing the placement of a central venous catheter, an advanced practical skill according to the classification used in our review, Peyton’s Step 3 – the student explains each sub-step while the teacher follows the student’s instruction – was identified to play the most crucial role in contributing to students’ learning success [24]. However, Peyton’s method of instruction was designed for a 1:1 teaching which does not reflect the typical learning situation. In skills labs, mostly small group teaching takes place with the ideal instructor : student ratio for teaching suturing skills having been identified to be 1:4 [7]. In a descriptive study on skills lab training for the same advanced practical skill mentioned above, central venous catheter insertion, a modified Peyton’s Four-Step Approach for small group teaching was described as being well accepted by students and easy for instructors to realize [29]. Whether this approach works equally well for basic practical skills and leads to similar or improved learning has not been described yet.

One limitations of our study is that many different search terms had to be used because “basic practical skills” as they were defined for our study are included in several other terms in the medical education literature and the list of search terms might still not have been complete. The focus of our review was on trying to summarize the evidence for how to teach basic practical skills in undergraduate medical education with the best effects. Hence, we only chose controlled studies which is a strength of our study. A weakness is, however, that we neglected to appraise the quality of the individual studies included in this review. Furthermore, despite individual assessment of all included papers by both authors, we might have been subject to errors during the data extraction and analysis and there also might have been a risk of reporting biases. Since the main focus of our study was on evidence-based teaching and learning methods in undergraduate medical education, we did not take influences of cognitive psychology and training of instructors into account as suggested influences were only found in manuscripts having been assigned to other categories. In addition, effective teaching methods of teaching skills might also be extracted from studies in postgraduate medical education or from studies on teaching more complex practical skills which were not in the focus of this study.

In conclusion, our findings suggest that voluntary or obligatory self-study with multimedia applications like video clips of certain skills in combination with a structured program including the possibility for individual exercise with personal feedback by peers or teachers seems to provide a good learning opportunity for basic practical skills. Whether the combination of these different aspects of teaching which individually improve basic practical skills’ learning will lead to similar effects needs to be evaluated by further educational research.

## Competing interests

The authors declare that they have no competing interest.

## Figures and Tables

**Table 1:**
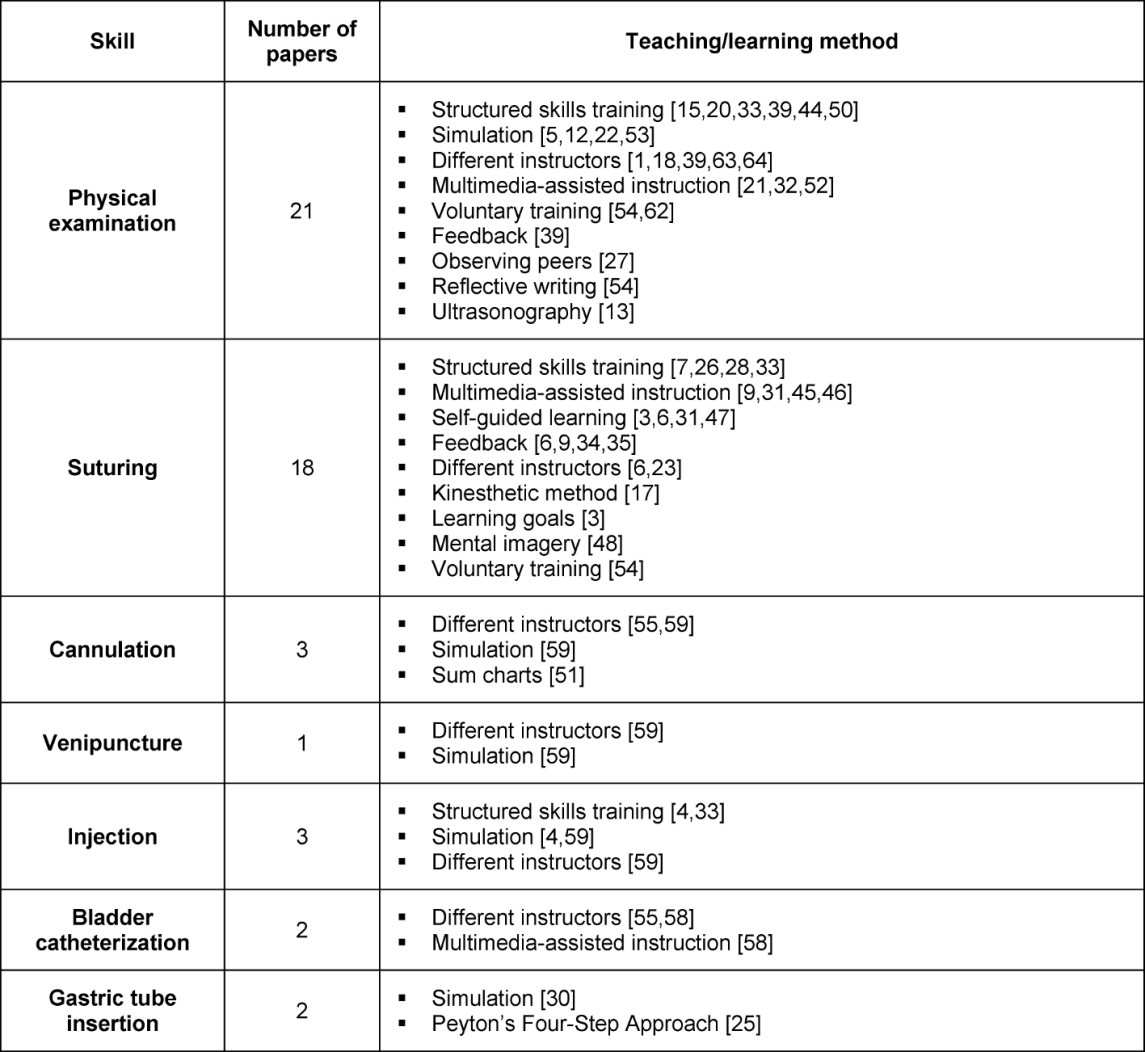
Teaching/learning methods for basic practical skills

**Table 2:**
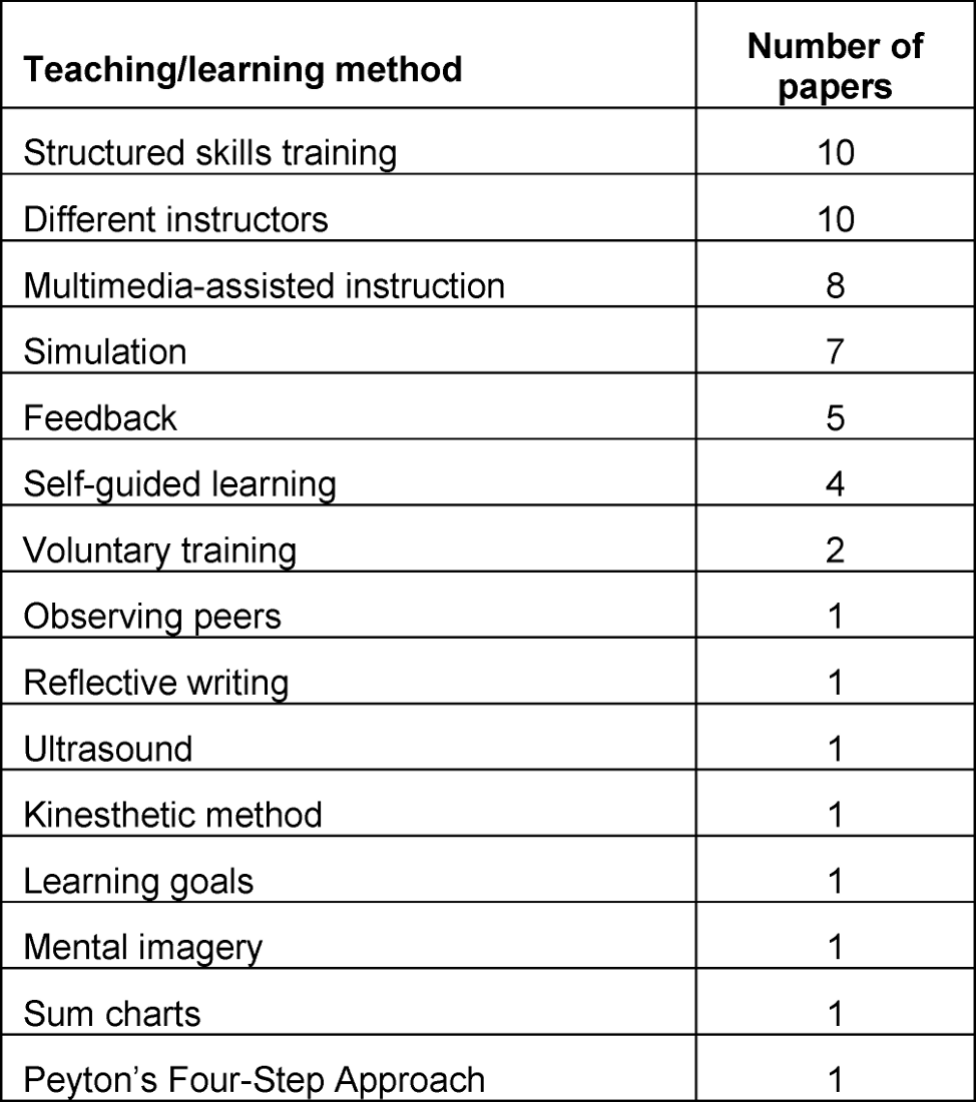
Number of papers identified per teaching/learning method
